# The Role of Amino Acids in the Crosstalk Between Mesenchymal Stromal Cells and Neoplastic Cells in the Hematopoietic Niche

**DOI:** 10.3389/fcell.2021.714755

**Published:** 2021-06-30

**Authors:** Martina Chiu, Giuseppe Taurino, Massimiliano G. Bianchi, Ovidio Bussolati

**Affiliations:** Department of Medicine and Surgery, University of Parma, Parma, Italy

**Keywords:** leukemia, glutamine, asparagine, mesenchymal stromal cell, bone marrow, arginine, tryptophan, amino acid transport system

## Abstract

Within the bone marrow hematopoietic cells are in close connection with mesenchymal stromal cells (MSCs), which influence the behavior and differentiation of normal or malignant lymphoid and myeloid cells. Altered cell metabolism is a hallmark of cancer, and changes in nutrient pools and fluxes are important components of the bidirectional communication between MSCs and hematological cancer cells. Among nutrients, amino acids play a significant role in cancer progression and chemo-resistance. Moreover, selected types of cancer cells are extremely greedy for glutamine, and significantly deplete the extracellular pool of the amino acid. As a consequence, this influences the behavior of MSCs in terms of either cytokine/chemokine secretion or differentiation potential. Additionally, a direct nutritional interaction exists between MSCs and immune cells. In particular, selected subpopulations of lymphocytes are dependent upon selected amino acids, such as arginine and tryptophan, for full differentiation and competence. This review describes and discusses the nutritional interactions existing in the neoplastic bone marrow niche between MSCs and other cell types, with a particular emphasis on cancer cells and immune cells. These relationships are discussed in the perspective of potential novel therapeutic strategies based on the interference on amino acid metabolism or intercellular fluxes.

## Introduction

Mesenchymal stromal cells (MSCs) are pluripotent stem cells able to differentiate into osteoblasts, chondroblasts and adipocytes. Within the bone marrow (BM), they constitute a quantitatively small population of pivotal importance for hematopoiesis (Li et al., 2016). MSCs secrete several factors that influence the behavior of either normal or malignant lymphoid and myeloid cells and can, in selected cases, significantly support the growth of malignant hematological cells. Moreover, a nutritional interaction and, possibly, a competition exist between cancer cells, MSCs and other cells of the BM niche. In this context, amino acids play a significant role in cancer progression and chemoresistance (Vettore et al., 2020). For example, MSCs secrete amino acids that support survival and drug resistance in leukemic blasts (Iwamoto et al., 2007; Zhang et al., 2012; Kwong-Lam and Chi-Fung, 2013; van Gastel et al., 2020). In other cases, cancer cells are extremely avid for a particular amino acid, so that the extracellular compartment is significantly depleted, influencing the behavior of MSCs in terms of either cytokine/chemokine secretion or differentiation ability. In addition, a direct nutritional interaction exists between MSCs and several types of immunity cells, which are themselves dependent upon selected amino acids for differentiation and activity. In this review, we discuss the enlarging role of the metabolic interactions between MSCs and hematological cancer cells by dissecting the role of selected amino acids in the bidirectional communications within the BM microenvironment.

## Arginine

Arginine (Arg, R) is a conditionally essential proteinogenic amino acid needed for several biological processes, such as protein synthesis, polyamine and nitric oxide (NO) production and urea cycle (Albaugh et al., 2017). The enzymes that mainly catabolize Arg are Arginases (ARG1 and ARG2) and Nitric Oxide Synthetases (NOS1-3) (McGaha et al., 2012) that convert Arg into ornithine (Orn) or citrulline (Cit), respectively. Arg is especially needed for T-cell activation, and its depletion cause T-cell anergy and dysfunction (Zea et al., 2004; Rodriguez et al., 2007). Indeed, one of the mechanisms elicited by MSCs to repress T-cell responses is the depletion of extracellular Arg, upregulating, either separately or in combination, ARG1 and iNOS (Bronte and Zanovello, 2005). A similar mechanism is exploited by acute myeloid leukemia (AML) primary blasts, which have an increased expression and activity of ARG2 (Mussai et al., 2013), thus favoring an immunosuppressive microenvironment. The peculiar arginine requirement of T-cell is also at the basis of the potential efficacy of PEGylated recombinant human Arginase I (PEG-Arg1), currently in clinical trials (Yau et al., 2015; De Santo et al., 2018b), in counteracting acute lymphoblastic T-cell leukemia (T-ALL) (Hernandez et al., 2010; Morrow et al., 2013; De Santo et al., 2018a).

The cytotoxic effects of PEG-Arg1 have been found neutralized by MSCs that can secrete citrulline, which is produced from ornithine through the enzyme Ornithine Transcarbamylase (OTC) (Kwong-Lam and Chi-Fung, 2013). In turn, the secreted citrulline is taken up by T-ALL blasts, which, subsequently, convert it into argininosuccinate and, eventually, into Arg trough the activity of Argininosuccinate Synthetase (ASS) and Argininosuccinate Lyase (ASL) (Sugimura et al., 1990).

## Asparagine and Aspartate

Asparagine (Asn, N) is a dead-end metabolite in humans since it cannot be further metabolized into aspartate (Asp, D). However, Asn is of pivotal importance for several processes since it is needed for CD8+T-cell activation (Hope et al., 2021; Wu et al., 2021), osteoblast differentiation (Chiu et al., 2020b), and vessel formation (Huang et al., 2017). The only enzyme able to synthetize Asn is Asparagine Synthetase (ASNS) that utilizes Asp for the carbon skeleton and the amino acid glutamine as the obliged nitrogen donor (Chiu et al., 2019). The majority of B-cell ALL blasts lack the expression of ASNS, are auxotroph for Asn and must take up the amino acid from the extracellular space. This auxotrophic phenotype is the rationale for the use of L-asparaginase, which rapidly hydrolyzes plasma Asn and, at a lower rate, glutamine, as a drug in ALL therapy. In combination with other drugs, such as vincristine and prednisone, asparaginase achieves a remission rate of more than 90% (Tabe et al., 2019). While there is no correlation between L-asparaginase resistance and ASNS gene induction in ALL blasts (Appel et al., 2006), it has been demonstrated a clear-cut protective, pro-leukemic role of MSCs, which secrete Asn rescuing Asn-starved blasts (Iwamoto et al., 2007). More recently, it has been observed a bidirectional crosstalk among leukemic and stromal cells upon L-asparaginase administration, with ALL blasts increasing Glutamine Synthetase (GS) expression and releasing glutamine, utilized by MSCs for the synthesis of Asn, which is then secreted to sustain ALL cell viability (Chiu et al., 2017). Interestingly, the fact that ASNS is upregulated in MSCs at diagnosis and does not further increase during L-asparaginase treatment (Dimitriou et al., 2014) suggests that MSCs must supply Asn to ALL blasts even before therapy, raising the possibility that Asn auxotrophy is somehow advantageous in the transformation pathway.

A somewhat similar behavior is observed in a murine model of AML, where MSCs fuel the synthesis of pyrimidine in leukemic blasts by producing Asp, thus supporting nucleotide biosynthesis and the resistance to cytarabine and doxorubicin chemotherapy (van Gastel et al., 2020).

## Cysteine

Cysteine (Cys, C) is crucial for cell redox balance, given its role as a substrate for the synthesis of the tripeptide glutathione (GSH), the most concentrated hydrophilic antioxidant in human cells. At neutral pH, Cys is unstable, and its thiol group spontaneously oxidizes thus producing cystine (Dewey and Beecher, 1965). However, while Cys uptake is mainly mediated by the ASC transporter systems, ASCT1 and ASCT2 (Kilberg et al., 1979), in most mammalian cells cystine influx is due to the antiport xCT, an heterodimeric transporter whose light chain is coded by *SLC7A11* gene, and occurs in exchange with intracellular glutamate (Kim et al., 2001). It has been shown that primary blasts of chronic lymphocytic leukemia (CLL), have an absent-to-low expression of xCT and, thus, lack a substantial device for cysteine import (Zhang et al., 2012). This behavior is the rationale for the use of Cyst(e)inase (Cystathionine-γ-lyase, Cystathionase, the enzyme that converts cystathionine derived from methionine into Cys) to control CLL growth (Cramer et al., 2017). Indeed, MSCs take up cystine, convert it into Cys and release the amino acid through the ASCT2 transporter to support GSH synthesis, relieve oxidative stress and favor chemoresistance in CLL blasts (Zhang et al., 2012). On the other hand, tumor associated MSCs are able to generate immunosuppression interfering with Cys metabolism of T-cells, that lack both xCT (Gmunder et al., 1991) and Cystathionase (Eagle et al., 1966), and require Cys supplementation from macrophages or dendritic cells (Gmunder et al., 1990). By secreting the immunosuppressive cytokine IL-10, MSCs repress the expression of Cystathionase in dendritic cells thus interfering with Cys supplementation needed by T cell for a full activation (Ghosh et al., 2016).

## Glutamine

Glutamine (Gln, Q) is a pleiotropic substrate needed for several metabolic reactions. Besides its role in protein synthesis, in several types of metabolically active normal and cancer cells Gln is utilized as an anaplerotic substrate to replenish TCA cycle with 2-oxoglutarate obtained from glutamate (Glu, E), through Glutamate Dehydrogenase (GDH) or one of the aminotransferases. In most cells, Gln is a major source of Glu by means of the activity of one of the glutaminases. Through glutamate, Gln sustains the synthesis of GSH (also fueled by cysteine/Glu exchange, see above) and several non–essential amino acids (NEAAs). Moreover, Gln is a precursor for nucleotides and glucosamine and behaves as a compatible osmolyte to maintain cell volume under hypertonic conditions (Chiu et al., 2020a). In addition, through its capability to interact with several active and exchange transporters, it is accumulated at high levels into the cell and can fuel by exchange the uptake of many NEAA and essential amino acids (EAAs). The main enzymes involved in Gln metabolism found dysregulated in cancers are glutaminases (GLS and GLS2, each with at least two isoforms) and Glutamine Synthetase (GS), the only mammalian enzyme which operates the endoergonic reaction between Glu and free ammonium to produce Gln (Mates et al., 2019).

Several hematological cancer cells rely on extracellular Gln supplementation for their growth. This is particularly evident for several kinds of AML blasts (Willems et al., 2013; Emadi et al., 2014; Jacque et al., 2015; Ni et al., 2019) and for multiple myeloma cells (MM) (Bolzoni et al., 2016; Giuliani et al., 2017; Thompson et al., 2017; Gonsalves et al., 2018, 2020), where GLS is usually up-regulated and GS down-regulated. Altered Gln metabolism has been also found in Del11q-positive CLL (Galicia-Vazquez et al., 2018), a deletion associated with poor-prognosis, and in Notch-driven T-ALL (Nguyen et al., 2021), where the up-regulation of Notch1 has been found associated with a GS downregulation. Consistently, an asparaginase preparation used in clinics, isolated from *Erwinia chrysanthemi* and endowed with higher affinity for Gln than that the enzyme from *E. coli* (Parmentier et al., 2015), has been found effective in counteracting both AML (Emadi et al., 2018, 2020) and multiple myeloma growth (Bolzoni et al., 2016; Soncini et al., 2020).

Adipocytes, one of the BM cell populations that can derive from MSCs, are able to counteract l-asparaginase effects on ASNS-positive ALL cells by providing Gln, newly synthetized by GS (Ehsanipour et al., 2013). Although the effects of Gln depletion on adipogenic differentiation have not been investigated, it is known that obesity implies poor prognosis also in childhood ALL (Butturini et al., 2007).

In the case of myeloma, besides being an Achille’s heel potentially targetable in clinics, the huge consumption of Gln by cancer cells causes a partial depletion of the amino acid in the BM, where Gln concentration decreases *in vivo* from 0.6 to 0.4 mM (Bolzoni et al., 2016). This change triggers a nutritional competition with nearby cells that may have heavy consequences. For instance, it severely impairs MSCs differentiation into osteoblasts (Chiu et al., 2020b), since Gln is required to sustain ASNS-dependent Asn synthesis during osteoblastogenesis (Yu et al., 2019; Shen et al., 2021), thus potentially contributing to the osteolytic bone disease characteristic of the disease. On the other hand, Gln depletion it is known to up-regulate GS in mesenchymal and immune cells, where GS induction leads to M2-like macrophages polarization (Palmieri et al., 2017; Menga et al., 2020). If this would occur in myeloma, the consequent immunosuppression should be sensitive to GS inhibition, which has been demonstrated in murine models to skew macrophages toward M1-like polarization (Palmieri et al., 2017; Menga et al., 2020). Although this issue awaits further investigation, it is interesting that Gln restriction in differentiating MSCs changes the expression of cytokines and chemokines involved in monocyte recruitment (Chiu et al., 2020b).

## Tryptophan

Tryptophan (Trp, W) is an essential amino acid and its availability is controlled by absorption and degradation, mainly due to Indoleamine 2,3-dioxygenase (IDO), which converts the amino acid into kynurenine (Badawy, 2017). IDO is expressed in antigen presenting cells (APCs) and was firstly described to promote immunotolerance during pregnancy to avoid fetal rejection (Munn et al., 1998). Interferon-γ increases IDO expression also in bone marrow MSCs, which, hence, hinder T cell responses (Meisel et al., 2004; Aggarwal and Pittenger, 2005; Liang et al., 2018), a mechanism crucial to promote immunosuppression. Indeed, both Trp depletion and kynurenine are able independently to cause T-cell anergy and trigger apoptosis (Platten et al., 2012). This mechanism has been exploited also by cancer cells given that MSCs within the tumor microenvironment express IDO and reduce CD8+T cell infiltration (Ling et al., 2014). Moreover, kynurenine is able to skew the differentiation of T-cells to Treg cells (Nguyen et al., 2010), thus further favoring tumor immune evasion. A positive correlation has also been found between IDO expression in AML and Treg induction (Wells et al., 2021). While clinical trials of IDO inhibitors in patients with solid cancers, although well-tolerated, have been dismissed given their unclear benefit (Jung et al., 2019; Long et al., 2019; Reardon et al., 2020), these drugs have been found promising for myelodysplastic syndrome (Komrokji et al., 2019), and a specific trial for AML is ongoing (NCT02835729) (Wells et al., 2021), opening new perspectives for its exploitation in hematological cancers.

## Conclusion

**FIGURE 1:**
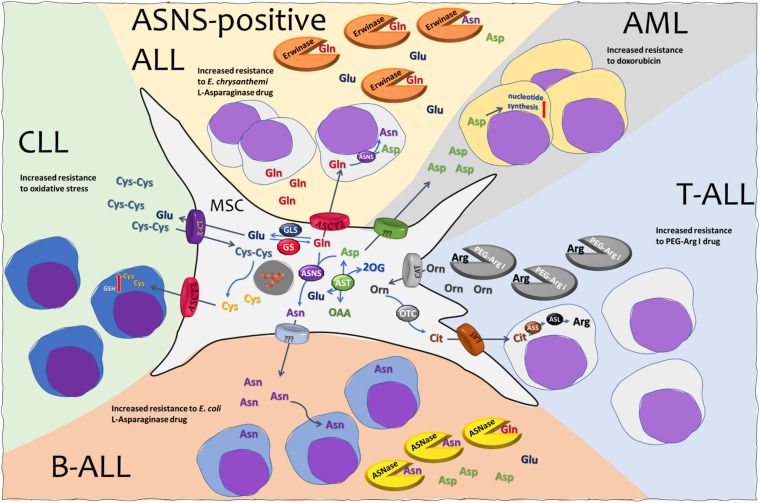
Mesenchymal stromal cells (MSCs) and amino acid crosstalk in leukemia. See text for explanation. MSC, mesenchymal stromal cell; ALL, acute lymphoblastic leukemia; AML, acute myeloid leukemia; CLL, chronic lymphocytic leukemia; ASNS, asparagine synthetase; ASL, argininosuccinate lyase; ASS, argininosuccinate synthetase; AST, aspartate aminotransferase; OTC, ornithine transcarbamylase; GLS, glutaminase; GS, glutamine synthetase; ASNase, l-asparaginase from *E. coli*. Erwinase, l-asparaginase from *E. chrysanthemi*; PEG-Arg I, PEGylated recombinant human arginase I; ASCT2, alanine serine cysteine transporter 2; CAT, cationic amino acid transporter; LAT, large neutral amino acid transporter; X-CT, cystine/glutamate transporter.

In this review we recapitulated the involvement of some amino acids in the complex cross-talks existing among hematological cancer cells and MSCs within the BM context (Figure 1). Drugs producing a selective amino acid depletion seem promising for the control of several kinds of hematological cancers, either auxotrophic for a given nutrient or requiring large amounts of a given amino acid. Moreover, metabolic interactions between MSCs and hematological cancer cells, as well as immune system cells, are often complex and should be taken into account to avoid or counteract possible protective activities during chemotherapy. Several approaches aimed at blocking the trophic activities of MSCs on blasts appear worthy of future investigations, such as, for example, GS inhibitors for ALL (Ehsanipour et al., 2013; Chiu et al., 2017), OTC inhibitors for T-ALL (Kwong-Lam and Chi-Fung, 2013) and Cyst(e)inase for CLL (Cramer et al., 2017). On the other hand, MSCs may be also targeted to mitigate tumor-induced immunosuppression, blocking Arg or Trp depletion by stromal cells (Bronte and Zanovello, 2005; Platten et al., 2012). Exploiting drugs involved in amino acid metabolism, cancer-independent effects of amino acid depletion on MSC population themselves should also be considered, given, for example, the specific requirement for Gln and Asn during MSC differentiation into osteoblast (Chiu et al., 2020b). Thus, an intense experimental work on the biology of both the normal and the neoplastic BM niche will be necessary to achieve successful combined therapies of hematological cancers, based on metabolic suppression and specific amino acid supplementation.

## Author Contributions

All authors contributed to the article and approved the submitted version.

## Conflict of Interest

The authors declare that the research was conducted in the absence of any commercial or financial relationships that could be construed as a potential conflict of interest.
